# 198. Comparison of Antibiotic Treatment Durations for Symptomatic Bacteriuria in Veterans with Spinal Cord Injuries or Disorders (SCI/D)

**DOI:** 10.1093/ofid/ofab466.198

**Published:** 2021-12-04

**Authors:** Michelle Fang, Scott T Johns, Ariel Ma

**Affiliations:** 1 VA San Diego Healthcare System, San Diego, CA; 2 San Diego VA Healthcare System, San Diego, California; 3 VA San Diego Medical Center, San Diego, California

## Abstract

**Background:**

It is unclear based on published literature whether shorter courses of antibiotic treatment may be appropriate for urinary tract infections (UTI) in patients with SCI/D given their complex baseline clinical status.

**Methods:**

This retrospective cohort study was conducted at the VA San Diego Healthcare System (VASDHS), which has a dedicated SCI/D unit. Adults with SCI/D were identified for inclusion if they had received antibiotics for a positive urine culture in conjunction with UTI symptoms from 1/2018-12/2020. Individual UTI events were excluded if associated with potential sources of harbored infection, anatomic abnormalities increasing risk of bacteriuria, non-bacterial pathogens, concurrent infections prolonging antibiotic treatment, or antibiotic courses managed outside of VASDHS. Treatment groups comprised UTI events treated with no more than 7 days of antibiotics (group 1) versus more than 7 days (group 2). Study endpoints were recurrence or new incidence of UTI within 30 and 90 days after completion of antibiotic treatment and onset of *C. difficile* infection or death within 30 or 90 days, respectively, after treatment completion. Statistical tests included Chi-square, Mann-Whitney U, and logistic regression.

**Results:**

One-hundred and seven patients with 241 unique UTI events were included in this study, with 79 events in group 1 and 162 events in group 2. Baseline characteristics were similar across both groups, aside from a higher incidence of hospital admission and more severe SCI/D based on the American Spinal Cord Injury Association (ASIA) impairment scale in group 2. Efficacy outcomes are described in Table 1. No deaths occurred within 90 days of treatment completion, and *C. difficile* infection occurred in 1 patient in group 2 after 3 days of antibiotic therapy. Duration of antibiotic therapy was not predictive of treatment failure within 30 days of antibiotic completion. Factors predictive of treatment with longer courses of antibiotic therapy included hospital admission and more severe ASIA impairment scale score.

Table 1. Incidence of UTI after antibiotic completion

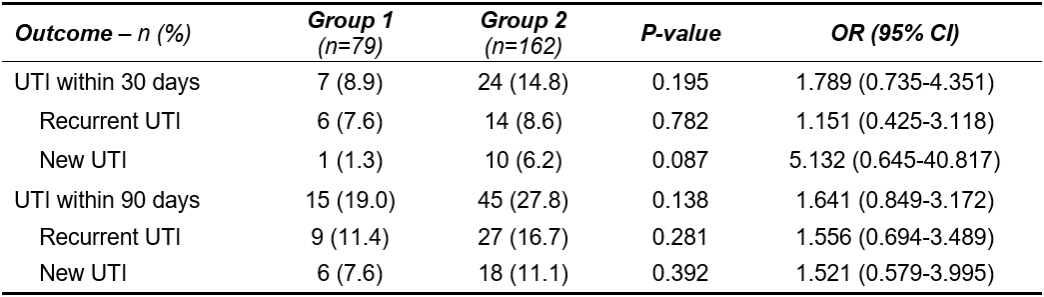

**Conclusion:**

The findings of this study suggest that for some patients with SCI/D, UTI treatment lasting 7 days or fewer may be effective compared to longer courses of antibiotics and could be beneficial in reducing collateral damage from antibiotic use.

**Disclosures:**

**All Authors**: No reported disclosures

